# Development and pilot of a framework to evaluate reproductive health call centre services: experience of Marie Stopes international

**DOI:** 10.1186/s12913-015-1064-0

**Published:** 2015-09-21

**Authors:** Pallavi Yagnik, Judy Gold, Mark Stoove, Barbara Reichwein, Caroline van Gemert, Nick Corby, Megan S C Lim

**Affiliations:** Centre for Population Health, Burnet Institute, Melbourne, Australia; Marie Stopes International, London, UK; Department of Epidemiology and Preventive Medicine, Monash University, Melbourne, Australia; Burnet Institute, 85 Commercial Rd, Melbourne, 3004 Australia

**Keywords:** Call centre, evaluation, monitoring and evaluation, framework, guidelines, reproductive health, abortion, service provision

## Abstract

**Background:**

Call centres can improve the effectiveness of health services by helping reduce access barriers associated with stigma and geography. This project aimed to develop and pilot a standardised evaluation framework to assess Marie Stopes International reproductive health call centres.

**Methods:**

Consultations were held with staff from the 14 existing international call centres to gauge current monitoring and evaluation processes, identify gaps, and establish evaluation needs. The draft framework was then piloted in the Marie Stopes Mexico call centre using client and provider surveys, mystery callers and a review of call centre records.

**Results:**

A flexible framework was developed to allow call centres to measure the effectiveness of services offered. Nineteen indicators were developed to assess access, equity, quality and efficiency. The pilot found pre-defined ranges for indicators of access were not appropriate for a high-functioning call centre that was already achieving nearly 100 % compliance. Several indicators could not be measured due to a lack of routine data collection systems.

**Conclusions:**

A standardised evaluation framework will allow comparisons over time and between call centres in different countries. Future assessments could be improved by establishing routine, reliable data collection systems prior to framework implementation. This is one of the first attempts to standardise the evaluation of a reproductive health call centre and establishes a method by which they can be monitored, and thus improved, over time.

## Background

Information and communication technologies (ICTs) are increasingly being used by health services to improve health care delivery and benefit clients and healthcare professionals n. eHealth - the use of ICT for health – could transform delivery of health services in developing countries by increasing access to information and services. eHealth platforms can also reduce barriers associated with stigma and discrimination that exist for face-to-face service provision, particularly for socially sensitive health issues (e.g., sexual and reproductive health) and where cultural mores impinge on service access [1–3].

Telephone call centres, one eHealth approach, are increasingly being used as a core component of health service delivery in developing countries [4]. Call centres share many of the advantages that underpin general eHealth approaches to health service provision in developing countries by increasing both geographic and socio-economic accessibility of health advice and information [4, 5] and offering clients anonymity that may encourage more frank and open discussion of sensitive health matters [3, 6, 7]. Although a review of health call centres in developing countries described several such services reaching millions of people, the review also identified a lack of evaluation of these services [5]. The limited evaluations undertaken have typically utilised only call tracking data or caller satisfaction surveys. The only published call centre evaluation from a developing country (Democratic Republic of Congo), reported few indicators including call volume, gender and province of caller, and reason for call [8].

Marie Stopes International (MSI) is one of the largest global providers of sexual and reproductive health services; in 2011, 14 MSI country programmes had established call centres. These call centres operate in diverse settings (Africa and the Middle East, Asia and the Pacific, Latin America and Europe), provide a variety of services (information provision, service referral, appointment booking, follow up and support), use an array of ICT (landline and mobile phones, text messaging, online chat) and have call volumes ranging from 80 to 27,000 per month [9]. There is no standardised data collection and evaluation methodology for the call centres – some use paper based records and others electronic systems, with data recorded at call, and sometimes individual client, level.

To ensure effectiveness of services provided, it is important that the quality and efficiency of call centres are monitored and evaluated using a rigorous approach. However, despite the increasing popularity of call centres globally there remains no agreed set of indicators to evaluate call centre performance in any setting. In addition, there is limited published evidence for the methodology of developing and implementing service evaluations for organisations that operate globally with diverse population groups.

This study had two aims; 1) to develop and test a standardised MSI Call Centre Evaluation Framework (the Framework) and 2) to evaluate the effectiveness of an MSI call centre in the middle income setting of the Marie Stopes Mexico call centre (MSMx) using the Framework. The results presented here describe learnings from the pilot implementation of the Framework at MSMx, in order to inform similar evaluations internationally.

## Methods

### Framework development

Marie Stopes International contracted the Burnet Institute (Australia) to develop the Framework and conduct the Framework pilot. The Framework was developed during 2011 using an iterative ‘action research’ approach’ (i.e. developed over time based on the emerging findings), via online, phone and in person consultations with Marie Stopes call centre staff from the then existing 14 call centres. Consultations consisted of a baseline needs analysis questionnaire emailed to all 14 representatives, followed by on-going consultation to;

- Map and identify the indicators already being measured at each call centre;
- Map and identify the data collection systems in place at each call centre;
- Identify country specific elements that need to be considered in a framework, (e.g. cultural norms and attitudes); and
- Provide broad direction for the development of the Framework.

A scoping visit to the nominated pilot site in Mexico City (MSMx) was conducted by a Spanish-speaking external evaluation consultant (PY) in December 2011. The scoping visit sought to establish the context of operations to inform how a set of indicators could be framed and implemented; what monitoring and evaluation (M&E) systems were in place; what new systems could practically be established; how the call centre service was delivered, including the resources and support provided to call operators; and build relationships with call centre staff to facilitate the piloting of the Framework in the call centre.

### Pilot evaluation in Mexico

The draft framework was tested in a pilot evaluation of MSMx in April 2012. At the time, MSMx operated four clinics in Mexico City and two clinics in the Southern state of Chiapas; clients could make appointments for MSMx services by either calling the call centre or attending the clinic. In 2012, Mexico had on average 17.0 fixed line telephone subscriptions and 83.3 mobile subscriptions per 100 inhabitants; 40 % of individuals used the internet in the past 12 months; this is similar to overall global averages of 16.7, 88.1, and 35 %, respectively [10].

The MSMx pilot evaluation included data collection from Mexico City service providers at all four clinics (via focus groups and interviews), callers to the call centre (via online survey), clients attending one of the Mexico City clinics (via in-person interviews), mystery callers (non-call centre MSMx staff), and review of routinely collected financial and electronic call data. Data from all sources were collated and reported against the relevant indicators in the evaluation framework. Indicators that required comparison over two time periods were compared between April 2010 and March 2011 (2010/11) and April 2011 and March 2012 (2011/12).

All data collection was completed in Spanish; results were translated by author PY into English. Quotes indicative of the majority sentiment were selected for inclusion by the author PY. All participants provided verbal or written informed consent.

Ethical approval for the pilot evaluation was obtained from The Alfred Hospital Human Research Ethics Committee.

## Results

### Framework development

An representative from 12 out of the 14 MSI call centres (86 %) completed the online questionnaire, which revealed a variety of current M&E practices (Table 1). In all but one country, very few monitoring indicators were currently used. Three countries reported that they had not yet put in place any monitoring frameworks.

**Table 1:** Results of survey of MSI call centres existing evaluation practices

		Number of call centres (N = 12)
Indicators currently utilised	Call numbers/tracking	8
	Service bookings/referral rates	9
	Nature of call	4
	Client feedback	2
Evaluation techniques currently utilised	Assess call centre operators	5
	Mystery client callers	4
	Phone interviews	2
	Client satisfaction	4
	Call database analysis	4
	Call centre staff feedback	3
	Informal client feedback	1

To ensure the Framework reflected MSI’s organisational objectives, a set of indicators were developed through review of existing call centre practice, compilation of current indicators used by call centres and other MSI services, and consultation with call centres. Nineteen indicators were chosen and categorised under the existing MSI M&E key areas: (Table 2) [11].

**Table 2:** MSI Call Centre Evaluation Framework

INDICATOR TYPE: [U]= Universal, [R]= Best practice- highly recommended, [O]= Best practice- optional				
Indicator	Recommended data source	**Optimal suggested range	Satisfactory suggested range	Bare minimum suggested range
*Access*				
A.1 [U] The number of calls received have increased by x% since y year	Call records	21-30%	11-20%	0-10%
A.2 [R] The percentage (%) of calls converted into appointment bookings have increased by x% since y year	Call records	20-30%	10-20%	0-10%
A.3 [R] The percentage (%) of bookings converted into attended appointments have increased by x% since y year	Call records	31-40%	21-30%	0-20%
A.4 [O] The percentage (%) of clients in MSI clinics referred by call centre has increased by x% since y year	Clinic records	21-30%	11-20%	0-10%
A.5 [O] The number of referrals made to other services has increased by x% since y year	Data analysis	10-15%	5-10%	0-5%
Equity				
A.6 [U] The percentage (%) of potential clients who are young people^1^ has increased by x% since y year.	Call records	10-15%	5-10%	0-5%
E.1 [R] The percentage (%) of potential clients who are from [particular target group of interest] has increased by x% since y year	Call records	10-15%	5-10%	0-5%
E.2 [R] The percentage (%) of potential clients who live below the poverty line has increased by x% since y year	Call records	10-15%	5-10%	0-5%
E.3 [O] The percentage (%) of new potential users of family planning^2^ has increased by x% since y year	Call records	10-15%	5-10%	0-5%
*Quality: Outputs*				
Q.1 [U] x% of “mystery” client callers are satisfied with MSI call centre service	Mystery caller report	81-95%	66-80%	50-65%
Q.2 [R] x% of callers are satisfied with MSI call centre service	Client survey/ interviews	81-95%	66-80%	50-65%
Q.3 [O] x% of callers report to be enabled and encouraged to participate effectively in their own care or treatment	Client survey/ interviews	81-95%	66-80%	50-65%
Q.4 [R] x% of service providers are satisfied with MSI service delivery due to call centre	Provider survey/ focus groups	81-95%	66-80%	50-65%
*Quality: Process*				
Q.5 [U] % of calls answered within 15 seconds	Call records	81-90%	71-80%	60-70%
Q.6 [U] The average call abandoned per year is less than x% *(standard is 3%)*	Call records	1-5%	5-10%	10-15%
Q.7 [R] The average level of ‘call quality’ of call centre staff has increased to x% since y year	Call Quality tool	81-95%	66-80%	50-65%
Q.8 [O] The percentage (%) of call centre staff that report that training and resources are adequate has increased to x% since y year	Staff survey/ interviews	81-95%	66-80%	50-65%
F.1 [U] The average cost per call has reduced by x% since year y	Call and HR records	10-15%	5-10%	0-5%

^1^The MSI definition for youth of interest is adolescents (aged 15-19 years) but in the case that a country does not have this breakdown available, they can use the most appropriate data

^2^“New potential users of family planning” refers to call centre callers who are not currently using a modern method of family planning

**Targets for indicators should never be set at 100%, as it is very difficult to guarantee being able to measure 100% in a survey. This is because there is scope for error in research

Please note that over subsequent evaluations, suggested ranges should be revised to encourage service improvements in your call centre. In doing this you may like to seek advice from your National Research Manager and/or the Regional Research Advisor

- *Access:* every potential client can easily reach or obtain MSI services regardless of financial, geographical and/or cultural barriers.
- *Equity*: every potential client has an equal opportunity to obtain MSI services and products regardless of their socio-economic status.
- *Quality*: All MSI services and products meet high clinical standards and quality of care that is client focused and responsive to client needs.
- *Efficiency:* every MSI programme produces the maximum possible output from a given set of inputs, thus being cost effective and sustainable.

Indicators were classified based on their relevance to all call centres:

- *Universal indicators* are vital to the functioning of all MSI call centres, and hence should be measured by all call centres with small to large scope and capacity. These indicators are all necessary, feasible or within a high priority group, identified by MSI.
- *Best practice - highly recommended* indicators are strongly encouraged to be measured.
- *Best practice - optional* indicators are encouraged to be measured, although not necessary for all call centres given the variation in services provided, and range in resources and capacity available to conduct call centre evaluations.

For each indicator, suggested *Minimum*, *Satisfactory* and *Optimal* ranges were provided, to reflect the level at which the call centre is operating against any given indicator. Using these ranges, a call centre evaluation can benchmark call centre effectiveness based on whether it is operating at a minimum, moderate or high level for the respective indicator. Suggested ranges were based on intervals identified in stakeholder consultations with MSI staff during the development of the framework. Hence, it should be noted that there were limited data to validate these suggested ranges.

Although the framework indicators were quantitative, the framework recommended that additional qualitative methods of data collection (e.g. service provider and client interviews) be considered to add further depth to the evaluation of call centres.

### Pilot evaluation in Mexico

#### Response rate

Of the 2520 MSMx callers sent email invitations, 191 completed the questionnaire (response rate 7.6 %). Clients attending appointments at MSMx clinics were invited by MSMx clinical staff to participate in interviews, of which ten were interviewed in-person. Four of eight doctors invited to complete the service provider questionnaire (50 %) participated. Thirty of 40 (75 %) MSI nurses and reception staff participated in four focus groups.

#### Access

Results, based on call records shown in Table 3 demonstrate that the call centre improved in terms of access between 2010–11 and 2011–12 by increasing call volume by 28.5 % and increasing the percentage of calls converted into appointment bookings (an increase of 23.5 %), the percentage of attended appointments (an increase of 0.5 %) and referrals to clinics by the call centre (an increase of 1.1 %). The percentage change between 2010–11 and 2011–12 met the optimal target range for indicator A1, the bare minimum range for indicators A2, A3 and A4.

**Table 3:** Results of Access and Quality indicator data for from call and clinic records for MSI Mexico call centre, April-March 2010–11 and 2011-12

	Indicator	2010-11	2011-12	% change
N Calls answered	A1	59505	76522	+22 %
N (%) Calls converted into bookings	A2	17050 (29 %)	21064 (28 %)	−1 %
N Total appointments booked		17389	21233	
N (%) Appointments made by clinic directly		339 (2 %)	169 (1 %)	
N (%) Of total appointments made by call centre	A4	17050 (98 %)	21064 (99 %)	+1 %
N (%) appointments attended	A3	12392 (71 %)	15728 (74 %)	+3 %
% of call answered within 15 seconds	Q5	94 %	92 %	
% Abandoned calls	Q6	5.7 %	5.0 %	

#### Equity

At the time of the evaluation, the call centre only had basic systems to collect routine data regarding socio-demographic characteristics of callers; therefore few data were available on equity indicators relating to the call centre’s reach of young people, minority groups and poverty status. The only characteristic collected routinely from the electronic call record systems was an aggregate age category, indicating 53 % of callers were aged less than 25 years.

#### Quality

Findings from mystery callers, online questionnaires and client interviews indicated high satisfaction levels with the call centre service among clients. The majority of client and mystery client satisfaction results reached or exceeded the optimal target range described in the Framework. For example, 96 % of clients surveyed online indicated that they were satisfied with the call centre experience. In the client quality indicator, (Q2) the ten interviewed clients agreed that the call centre service provided them with a high quality and accessible service.

Client interview: *“The call operator gave me a lot of useful information and I am very satisfied with it. He made me feel confident and safe. He gave me really good advice for whatever questions I had.”*

Client interview: *“The appointment and wait time were adequate. The operator was attentive. The information was useful. For example, they explained how the method of contraception worked and what it meant for my body.”*

Mystery client reports supported this result, with all mystery callers reporting a sensitive, empathetic, informative and responsive service.

Mystery client report: *“They were very kind, attentive and interested in helping. With a warm and caring voice they told me it was my decision to make and no one else’s.”*

Mystery client report: *“They were sensitive, as I can’t get out of work easily and they gave me other hours to access the services.”*

Service providers were also satisfied with the call centre service and reported that the call centre was increasing their capacity to provide a higher quality service to clients. However, key messages emerging from focus groups including the need to develop more rigorous feedback channels between the call centre and the clinics; ongoing training and support for call operators; and the need for call operators to provide more succinct information to clients regarding medical procedures and punctuality. The focus groups undertaken in this evaluation were the first time service providers had been asked to provide feedback on the call centre service, which they reported valuing greatly.

The analysis of process indicators also provided positive insights into the operation of the call centre, with call wait time for almost all calls well within the target of 15 seconds (94 % in 2010–11 and 94 % in 2011–12). Call abandonment rates were slightly higher than the target of 3 %, but appeared to be reducing over time (5.9 % in 2010–11 to 5 % in 2011–12).

#### Efficiency

The average cost per call in 2011 was 8.20 Mexican Pesos (approximately GBP 0.38/USD 0.59).

## Discussion

This project aimed to develop and pilot a call centre evaluation framework. While there are many emerging health call centres in developing countries, none have reported methodologies or results of monitoring and evaluation in any detail [5, 8]. The development and piloting of this framework provides a useful model for other organizations operating health related call centres considering how to better monitor and evaluate their operations.

### Performance of Mexico call centre

Through pilot testing of the Framework, the Mexico City call centre was found to be operating very effectively with regards to access, quality and efficiency, however insufficient data were collected to measure most equity indicators. The results indicated that between 2010–11 and 2011–12 the call centre was able to attract a growing number of clients to MSMx services over time and maintained the percentage of calls converted into appointment bookings, the percentage of attended appointments and referrals to clinics by the call centre.

The call centre operated at a high level; findings demonstrated that the call centre was able to make bookings and engage clients despite a large increase in calls. Clients and mystery clients echoed these results, with the majority reporting high satisfaction with the booking system, manner and attitude of call operators, the quality of information provided by call operators and the level of organisation of the call centre. Most quality indicators fell within the optional target range.

Although the MSMx clinics and call centre already work in an integrated manner, service providers identified that communication can be improved. Integrated health services, characterised by a high degree of communication and collaboration among health professionals; are thought to be more likely to offer a higher quality, more effective and more efficient service [2]. Focus groups revealed the value of holding discussions with service providers, as the focus groups undertaken in this evaluation were the first formal opportunity for service providers to provide feedback on the call centre service. Increasing the communication between the two aspects of MSIs service delivery will assist in bridging any service gaps or shortfalls. Suggestions for feedback and communication included: peer swaps, joint meetings, or joint training between call centre and clinic staff.

The findings of the pilot evaluation at MSMx should be interpreted with consideration of the limitations of the evaluation framework discussed below.

### Framework implementation

Our experience can be used by other non-commercial call centres to improve their monitoring and evaluation practices, particularly those with a focus on impact. There are limited evaluations of call centres internationally, most of which have used process evaluation methodologies only [8, 12] or review of staff recruitment and training methodologies [13, 14], and none of which had a focus on sexual health or women’s health, and only one was in a low-middle income country [8]. Further, there are no published evaluations of international organisations call centres operating in different countries and the process to develop systematic measures of comparison; this is a strength of this study.

The Framework was generally found to be suitable for evaluation purposes; however, some barriers to its implementation were noted. Addressing these barriers from the pilot evaluation is critical to the further refinement of the Framework for use by MSI Call Centres, and for other global organisations designing and implementing similar service evaluations.

Implementing an evaluation framework within a service setting involves several challenges, particularly in relation to programme capacity. A minimum level of evaluation skills is needed to implement the Framework to ensure robust, reliable and generalisable findings. The initial survey revealed low levels of M&E experience among MSI call centre staff; this may mean that staff training is required or that the evaluation is conducted by someone external to the local call centre team. Similarly, routine, ongoing data collection is necessary. In the Mexico call centre, although there were some call data available, there were limited data collected measuring equity, such as reach of poor, youth or minority groups. The survey of call centres revealed that most currently collect very minimal data routinely. Other call centres rely on manually recorded data, which may be difficult to analyse, or do not collect data routinely. For programming and future evaluation purposes, systems could be established to measure indicators either on routine basis from all callers or periodically from a subset. MSMx could also explore opportunities to utilise other research and surveillance data to better understand the needs to potential target populations, including those not currently accessing MSI services, to inform service delivery. Before conducting an evaluation using the framework, a rapid assessment of available data is recommended to ensure the scope of the evaluation is within the bounds of what can feasibly (and reliably) be assessed.

The pilot assessment included potential biases which may have skewed findings. For example, reports from mystery clients and service providers may have been subject to social desirability bias as they were MSI employees and thus may have felt obligated to provide positive results. Response rates to the online questionnaire were very low (less than 10 %); far lower than average rates reported in a review of telemedicine patient surveys in high income countries (all studies achieved a response rate above 50 %) [15]. This may have resulted in some sampling bias in relation to the clients that chose to complete online questionnaires; it is possible that those who felt more strongly about their experience (whether positive or negative) were more likely to respond. Operators may also have been less likely to invite those they thought would provide more negative responses. Clients might also have been concerned about providing negative feedback if they had an ongoing relationship with MSMx. Furthermore, the use of email as a methodology for inviting participation may have biased the sample towards those with greater access to the internet; however internet penetration in Mexico is a fairly high at 36.2 per 100 people in 2011, compared to other middle income countries [16], and as MSMx charges fees for service, it may attract clients from higher socio-economic groups. Despite the low response to email surveys, this was considered to be the only feasible method for large scale quantitative data collection as part of an ongoing evaluation framework. Email surveys were also considered more private than phone or postal surveys, an important consideration for this sensitive service. Future assessments could control for biases by implementing more rigorous sampling strategies and having sufficient resources to employ external mystery client interviewers.

The framework pilot highlighted a need for further review and revision of the suggested indicator performance ranges, to ensure they best reflect call centre performance. In the pilot it was found that some suggested ranges did not adequately reflect the performance level of the call centre. MSMx was performing at a very high level in relation to some access indicators, but saw a very small percentage increase between time periods, causing the indicator score to fall into the ‘bare minimum’ suggested range, despite the absolute value of the indicator being high (e.g. over 98 % of clients in clinics were being referred by the call centre (Indicator A4) in both time periods; a 0 % improvement). Caveats in relation to interpreting outcomes for call centres with very high performance levels need to be incorporated into the framework. In future, the framework could utilise data from newly established routine data systems and previous evaluations to set benchmarks. As more call centres implement the framework, target ranges may need to be shifted to best reflect call centre performance.

Although the collection of qualitative data is not essential to completing the framework, it is advisable for similar evaluations to include these. A strong message from focus groups in Mexico was the value of holding discussions with service providers, as the focus groups undertaken in this evaluation were identified as the first time they were asked to provide feedback on the call centre service. The focus groups of service providers identified suggestions to improve the call centre service which may not have been recognised otherwise. These suggestions are now being implemented in the call centre.

The framework was developed using an ‘action research’ approach, whereby involving all call centres in the development of the framework allowed them to have ownership over the process. This proved to be a very effective engagement technique and may increase uptake of the framework in the future. Although this approach may have taken longer to implement, the resulting product was a flexible, sensitive tool that could be used by call centres of any size. The ‘action research’ approach is recommended as a methodology for the development of similar evaluations.

## Conclusions

Health call centres have the potential to provide access to services to large numbers of people at a relatively low cost, avoiding some barriers of traditional services in terms of geography, accessibility, and privacy. However if call centres are poorly functioning, inequitable, or inefficient, this can lead to adverse health outcomes by missing an opportunity to reach underserved populations and wasting of scant resources. Thus systematic monitoring and evaluation of call centres is essential to ensure call centres are providing high quality services to those in need.

Through consultation and action research with MSI call centre staff in 14 countries, we developed a standardised call centre evaluation framework. The framework was piloted in an evaluation of the Mexico City call centre, and was found to operate effectively on the specified areas of access, quality and efficiency, with improvements noted for equity data. Additionally, the framework was found to be a suitable evaluation tool, with the proviso that staff implementing the framework have a minimum level of evaluation experience, that routine data collection systems are in place prior to implementation, biases are acknowledged and minimised, and that target ranges are routinely reviewed. Using a standard framework in the evaluation of call centres could improve their quality and lead to better services. We recommend this framework become routinely implemented in MSI call centres and that appropriate systems are established to measure it.

These findings are relevant for global organisations that are designing or implementing similar service evaluations, particularly as the establishment of more call centres in developing countries is likely with increased access to and decreased cost of telecommunications services. There is a notable lack of published work on the evaluation of call centres in both developed and developing country settings; this Framework and pilot evaluation provide a base which could be adapted for other settings and other types of call centres. In the future it is hoped that more of this type of evaluation will be conducted and shared, leading to the development of standardised indicators and evaluation frameworks which enable international comparison and improvement of call centres globally.

## Abbreviations

ICT: Information Communication Technology
M&E: Monitoring and Evaluation
MSI: Marie Stopes International
MSMx: Marie Stopes Mexico
